# Outcomes after SynCardia® temporary total artificial heart implantation: A 20‐year single‐center experience in 196 patients

**DOI:** 10.1111/aor.14860

**Published:** 2024-09-16

**Authors:** Artyom Razumov, Melchior Burri, Armin Zittermann, Darko Radakovic, Volker Lauenroth, Sebastian V. Rojas, Henrik Fox, René Schramm, Jan Gummert, Marcus‐André Deutsch, Michiel Morshuis

**Affiliations:** ^1^ Department of Thoracic and Cardiovascular Surgery, Heart and Diabetes Center North Rhine‐Westphalia Ruhr University of Bochum Bad Oeynhausen Germany; ^2^ Department of Cardiovascular Surgery, German Heart Centre Munich Technical University of Munich Munich Germany

**Keywords:** biventricular support, heart failure, total artificial heart

## Abstract

**Background:**

The SynCardia® temporary total artificial heart (TAH) serves as a mechanical circulatory support device for patients suffering from irreversible biventricular failure.

**Methods:**

This retrospective study analyzed 196 consecutive patients who underwent TAH implantation at our center from 2001 to 2021. We assessed survival rates and all‐cause mortality during TAH support, including survival post‐heart transplantation.

**Results:**

The median age of patients was 55 years, with 88% being male. The primary diagnoses included cardiomyopathy (43.9%), acute myocardial infarction (26.5%), and postcardiotomy heart failure (15.5%). At implantation, 87.2% of patients were classified as INTERMACS Profile 1. The median duration of support was 96 days (IQR: 23–227). Survival rates at 1, 6, and 12 months were 72%, 41%, and 34%, respectively. Postoperative rethoracotomy was necessary in 44.4% of patients; 39.3% experienced neurological events and 24.6% developed gastrointestinal bleeding. Overall, 64.8% of patients died while on support, primarily due to multiple organ failure (55.9%). Factors such as older age, higher bilirubin levels, postcardiotomy and specific underlying diagnoses were independent predictors of mortality during TAH support. On a positive note, 35.2% of patients underwent successful heart transplants, with 1‐, 5‐, and 10‐year posttransplant survival rates of 65%, 58%, and 51%, respectively.

**Conclusions:**

While high mortality rates persist among patients with biventricular failure, the SynCardia® TAH offers a viable interim solution for critically ill patients, particularly those who can be successfully bridged to heart transplantation.

## Graphical abstract

The SynCardia® TAH is the preferred device for biventricular support in patients unsuitable for other devices. Median support duration was 96 days, with survival rates of 72%, 41%, and 34% at 1, 6, and 12 months, respectively, and successful bridge to transplantation occurred in 35.2% of patients, with post‐transplant survival rates of 65%, 58%, and 51% at 1, 5, and 10 years, respectively. Given the extremely poor preoperative clinical status, a third of patients were successfully bridged to transplantation.

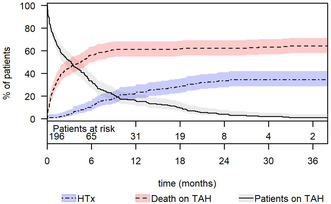

## 1 BACKGROUND

Various mechanical circulatory support devices and techniques are available for effective short‐ or long‐term circulatory assistance in patients with end‐stage heart failure.^1^ The development and availability of continuous‐flow left ventricular assist devices (LVADs) enable many patients with biventricular failure to be supported using LVAD‐based implantation strategies.^2^, ^3^ These include either an LVAD combined with temporary right ventricular extracorporeal life support devices or the biventricular placement of two LVADs. However, a subset of patients with acute or decompensated chronic irreversible biventricular heart failure have no alternative but to receive a total artificial heart (TAH) due to anatomical or technical constraints.^4^, ^5^, ^6^ At our institution, the SynCardia® temporary total artificial heart (SynCardia Systems Inc., Tucson, AZ, USA) has been the preferred device for supporting these critical patients since 2001. It is a pneumatically driven, pulsatile system utilized for the orthotopic replacement of both native ventricles, primarily serving as a bridge‐to‐transplant strategy.

Globally, over 2000 TAH implantations have been performed.^7^ About 2% of all long‐term mechanical circulatory support devices implanted worldwide are of this type. Recent years have seen a decline in the number of these implantations.^8^, ^9^ Data on outcomes following TAH implantation and subsequent heart transplantation post‐TAH are limited, particularly from European countries under the Eurotransplant donor organ allocation system. This study aims to provide updated insights into the use of this device at our center.

## 2 PATIENTS AND METHODS

### 2.1 Study design

Our study encompassed 196 consecutive patients who underwent SynCardia® TAH implantation at our institution from the initial procedure in February 2001 through December 2021. The primary objectives were to evaluate survival while on mechanical support and to identify predictive factors for mortality during TAH support. Secondary outcomes included the assessment of adverse events while under TAH support and survival following heart transplantation.

### 2.2 Data collection

This study received approval from the Institutional Review Board and Ethics Committee of the Ruhr University of Bochum, Germany (Approval No. N 2021–754). Because of the retrospective study design, requirement for written informed consent was waived. The study complies with the ISHLT Ethics statement. We retrospectively collected comprehensive data including baseline demographics, perioperative details, outcome data, and adverse events in a dedicated clinical database. Complete follow‐up data were available for all patients until either their demise while on mechanical support or following a heart transplant.

### 2.3 Patient selection

At our center, the SynCardia TAH is the preferred device for mechanically supporting critically ill patients with terminal, refractory biventricular pump failure. This applies particularly to patients in heart failure stage NYHA IV or INTERMACS levels 1–3, for whom alternative devices are unsuitable and those facing imminent death. We primarily utilize the TAH as a bridge to transplantation or bridge to candidacy. Prior to implantation, all patients undergo a thorough multidisciplinary evaluation, akin to the process for pretransplant patients before listing. Their INTERMACS profiles are assessed retrospectively, adhering to the guidelines and criteria outlined by Stevenson et al.^10^

### 2.4 Patient management

The techniques for implantation have largely remained consistent since the device's introduction.^11^, ^12^ Similarly, the postoperative management of patients, encompassing anticoagulation and antibiotic regimens, was conducted as described in previous reports.^13^ Patients who received the TAH were added to the cardiac transplantation waiting list once they had recovered and demonstrated normal organ function and overall health improvement.

### 2.5 Statistical analysis

Categorical variables were presented as absolute numbers and percentages. Continuous variables were expressed as means with standard deviations, or as medians with interquartile ranges (IQR), depending on their distribution as determined by the Shapiro–Wilk's test. The Student's *t*‐test was applied for comparing normally distributed continuous variables, while the Mann–Whitney *U* test was used for non‐normally distributed variables. Categorical variables were compared using Pearson's chi‐squared test or Fisher's exact test, as appropriate. We employed a competing risk analysis to construct the cumulative incidence rates of mortality on TAH, heart transplantation, and duration on mechanical support. Kaplan–Meier analysis was used to evaluate survival on the TAH leading to transplantation, as well as posttransplant survival, with the strata compared using the log‐rank test. For assessing survival on mechanical support, patients were censored at the time of transplantation. The Fine‐Gray method was utilized to analyze the impact of primary preoperative diagnosis on mortality rates while on TAH support and heart transplant rates. Primary diagnostic subgroups with a count of ≤10 were combined into a “Other indication” category to ensure comparability among groups. A multivariate Cox regression model was developed to identify independent predictors of death while under support. Variables with a *p*‐value ≤0.10 in the univariate analysis were included in the multivariate analysis. All statistical analyses were conducted using SPSS version 29 (Chicago, IL) and R version 4.3.1 (R Foundation for Statistical Computing, Vienna, Austria). A *p*‐value ≤0.05 was considered statistically significant in all analyses, and they were two‐tailed.

## 3 RESULTS

### 3.1 Patient population

All baseline characteristics of the patients are detailed in Table 1. The median age was 55 years (IQR: 46–61; range: 16–76), with a predominant male representation (173; 88.3%). At the time of TAH implantation, most patients were classified as INTERMACS Profile 1 (170; 86.7%) and Profile 2 (23; 11.7%). The median body mass index (BMI) was 26.3 kg/m^2^ (IQR: 23.6–29.3; range: 17.1–44.6). Preoperative median creatinine levels were 1.7 (IQR: 1.2–2.6; range: 0.3–10.9) mg/dL, and median total bilirubin levels were 1.64 (IQR: 0.90–3.66; range: 0.4–24.2) mg/dL. The 50 cc TAH model was implanted in six (3.1%) patients. The three leading causes of heart failure were cardiomyopathy (86 patients; 43.9%), acute myocardial infarction (52 patients; 26.5%), and postcardiotomy heart failure (31 patients; 15.8%; Table 2).

**TABLE 1:** Descriptive values of selected parameters recorded within 24 h before TAH implantation.

Variables	Overall group *n* = 196	Survivors under TAH support *n* = 69 (35.2%)	Died under TAH support *n* = 127 (64.8%)	*p‐*value
Demographic data				
Age (years)	55 (46–61)	51 (44–57.5)	58 (49–62)	***0.003***
Male gender, *n* (%)	173 (88.3)	63 (91.3)	110 (86.6)	0.33
Female gender, *n* (%)	23 (11.7)	6 (8.7)	17 (13.4)	
BMI (kg/m^2^)	26.3 (23.6–29.3)	26.2 (24.4–28.2)	26.8 (23–29.6)	0.730
Blood group, *n* (%)				
0	77 (39.3)	31 (44.9)	46 (36.2)	0.61
A	100 (51.3)	28 (40.6)	72 (57.1)	
B	15 (7.7)	9 (13)	6 (4.8)	
AB	4 (2.1)	1 (1.4)	3 (2.4)	
Blood laboratory parameters				
Serum protein (g/dL)	5.6 (1.1)	5.4 (0.9)	5.7 (1.2)	0.153
Urea (mg/dL)	69 (48–108.75)	65 (46–102)	71 (49–112)	0.355
Creatinine (mg/dL)	1.7 (1.2–2.6)	1.7 (1.2–2.9)	1.6 (1.2–2.4)	0.399
Total bilirubin (mg/dL)	1.64 (0.90–3.66)	1.61 (0.84–3.33)	1.7 (0.9–3.9)	0.606
AST (U/I)	76 (38–274)	103 (46–311)	66 (30–205)	***0.028***
ALT (U/I)	43 (19–120.75)	66 (27–173)	34 (18–96)	***0.039***
Gamma‐GT (U/I)	90 (44–184.50)	75 (37–158)	91 (49–204)	0.147
Alkaline phosphatase (U/I)	92 (68.5–136)	90 (69–115)	95 (68–147)	0.219
Lipase (U/I)	55.1 (23.8–146.5)	65 (24–160)	54 (24–138)	0.553
LDH (U/I)	532 (334.5–1340)	610 (437–1620)	451 (289–1140)	***0.007***
CRP (mg/dl)	10.7 (2.7–21)	10.9 (2.4–20.5)	10 (2.9–21.8)	0.597
Leukocytes (10^9^/L)	11.55 (8.725–16)	11.9 (9.5–15.4)	11.1 (8.1–16.1)	0.441
Thrombocytes (10^9^/L)	112 (59.75–183)	104 (68–181)	115 (57–183)	0.987
aPTT (s)	45 (36–56.75)	46 (37–57)	45 (34–57)	0.596
Prothrombin time (%)	68 (45.5–87.5)	67 (46–90)	68 (45–87)	0.775
Fibrinogen (mg/dL)	425 (295.25–533.5)	414 (295–508)	438 (303–550)	0.319
Antithrombin III (%)	71 (19)	71 (17)	71 (21)	0.960
Procedures before TAH implantation				
INTERMACS profile				
1	170 (86.7)	65 (94.2)	105 (82.7)	0.138
2	23 (11.7)	4 (5.8)	19 (15)	
3	2 (1)	0	2 (1.6)	
4	1 (0.5)	0	1 (0.8)	
Previous cardiac surgery, *n* (%)	129 (65.8)	43 (62.3)	86 (67.7)	0.447
Cardiopulmonary resuscitation, *n* (%)	98 (50)	40 (58)	58 (45.7)	0.100
Mechanical ventilation, *n* (%)	153 (78.1)	57 (82.6)	96 (75.6)	0.257
IABP, *n* (%)	105 (53.6)	51 (73.9)	54 (42.5)	***<0.001***
ECLS/ECMO, *n* (%)	93 (47.4)	37 (53.6)	56 (44.1)	0.202
VAD, *n* (%)	19 (9.7)	8 (11.6)	11 (8.7)	0.507

*Note*: Values are presented as median and interquartile ranges 25th–75th or *n* (%), except for serum protein and antithrombin III, presented as mean ± standard deviation.

Abbreviations: ALT, alanine aminotransferase; aPTT, activated partial thromboplastin time; AST, aspartate aminotransferase; BMI, body mass index; ECLS, extracorporeal life support; ECMO, extracorporeal membrane oxygenation; IABP, intra‐aortic balloon pump; INTERMACS, Interagency Registry for Mechanically Assisted Circulatory Support; LDH, lactate dehydrogenase; TAH, total artificial heart; VAD, ventricular assist device.

**TABLE 2:** Primary causes of heart failure.

Cause	*n* (%)
Cardiomyopathy	
Total	86 (43.9)
Ischemic cardiomyopathy	48 (24.5)
Dilated cardiomyopathy	31 (15.8)
Hypertrophic cardiomyopathy	6 (3)
Restrictive cardiomyopathy	1 (0.5)
Acute myocardial infarction	52 (26.5)
Postcardiotomy heart failure	31 (15.8)
Other indication	
Total	27 (13.8)
Acute myocarditis	10 (5.1)
Infective endocarditis	4 (2)
Cardiac graft rejection	7 (3.6)
Congenital cardiac disease	4 (2)
Primary pulmonary hypertension	1 (0.5)
Cardiac tumor	1 (0.5)

A comparison between patients who died while on TAH support and those who survived to heart transplantation revealed significant differences. Survivors were younger (median age 51 years [IQR: 44–57.5] vs. 58 years [IQR: 49–62], *p* = 0.003) and had a higher preimplantation requirement for intra‐aortic balloon pumps (73.9% vs. 42.5%, *p* < 0.001). Survivors also exhibited elevated levels of AST (103 U/L [IQR: 46–311] vs. 66 U/L [IQR: 30–205], *p* < 0.028), ALT (66 U/L [IQR: 27–173] vs. 34 U/L [IQR: 18–96], *p* < 0.039), and LDH (610 U/L [IQR: 437–1620] vs. 451 U/L [IQR: 289–1140], *p* < 0.007).

### 3.2 Outcomes

#### 3.2.1 Survival analysis

**FIGURE 1:**
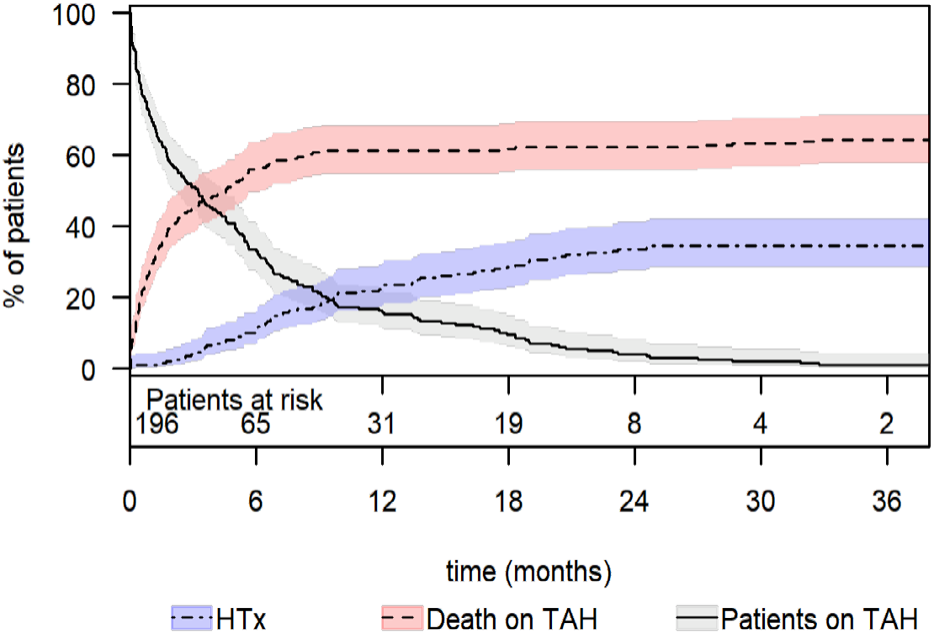
Competing risk analysis on the TAH in the overall cohort. 95% CI, confidence interval; TAH, total artificial heart. [Color figure can be viewed at wileyonlinelibrary.com]

**FIGURE 2:**
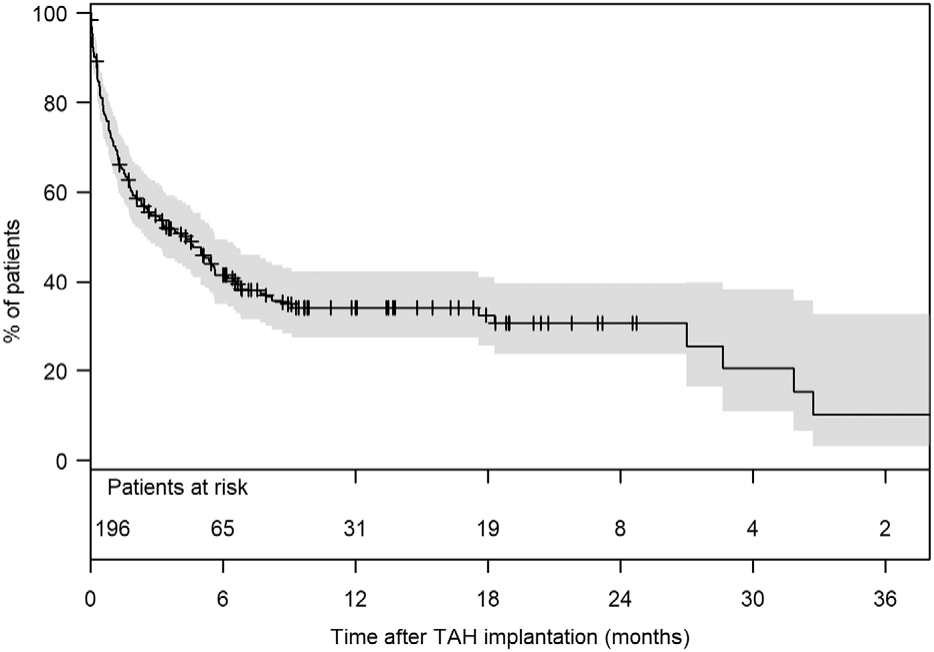
Survival on TAH (censored at heart transplantation). 95% CI, confidence interval; TAH, total artificial heart.

Competing risk analysis revealed that the cumulative incidence of mortality while on TAH support was 28% (95% CI: 22–35) at 1 month, 56% (95% CI: 50–64) at 6 months, and 61% (95% CI: 55–68) at 1 year (Figure 1). The cumulative incidence of heart transplantation while on TAH support was 1% (95% CI: 0.3–4) at 1 month, 11% (95% CI: 7–16) at 6 months, and 23% (95% CI: 18–30) at 1 year. The total support duration was 35.406 days (97 years). The median time under TAH support was 96 days (IQR: 23–227; range: 1–1611). Survivors had a significantly longer median support duration than non‐survivors (265 days [IQR: 152–472] vs. 37 days [IQR: 12–123], *p* < 0.001). The 1‐, 6‐, and 12‐month survival rates on TAH support were 72% (95% CI: 66–78), 41% (95% CI: 35–49), and 34% (95% CI: 28–42), respectively (Figure 2). A total of 127 patients (64.8%) died from multiple organ failure being the most common cause of death (71 patients; 55.9%; Table 3). The clinical signs of sepsis were observed in some patients with multi‐organ failure and mesenteric ischemia. However, nine (7.1%) patients died from pure sepsis. Among the patients who died, 57 (44.9%) had temporary MCS: 56 (44.1%) had ECLS/ECMO, and one patient had an Impella.

**TABLE 3:** Cause of death while on TAH.

Cause	*n* (%)
Multiple organ failure	71 (55.9)
Sepsis	9 (7.1)
Neurologic dysfunction	18 (14.2)
Pulmonary dysfunction	6 (4.7)
Mesenteric ischemia	12 (9.4)
Major bleeding	2 (1.6)
Device malfunction	4 (3.1)
Cardiac sarcoma	1 (0.8)
Suicide	1 (0.8)
Unknown	3 (2.4)

Abbreviation: TAH, total artificial heart.

**FIGURE 3:**
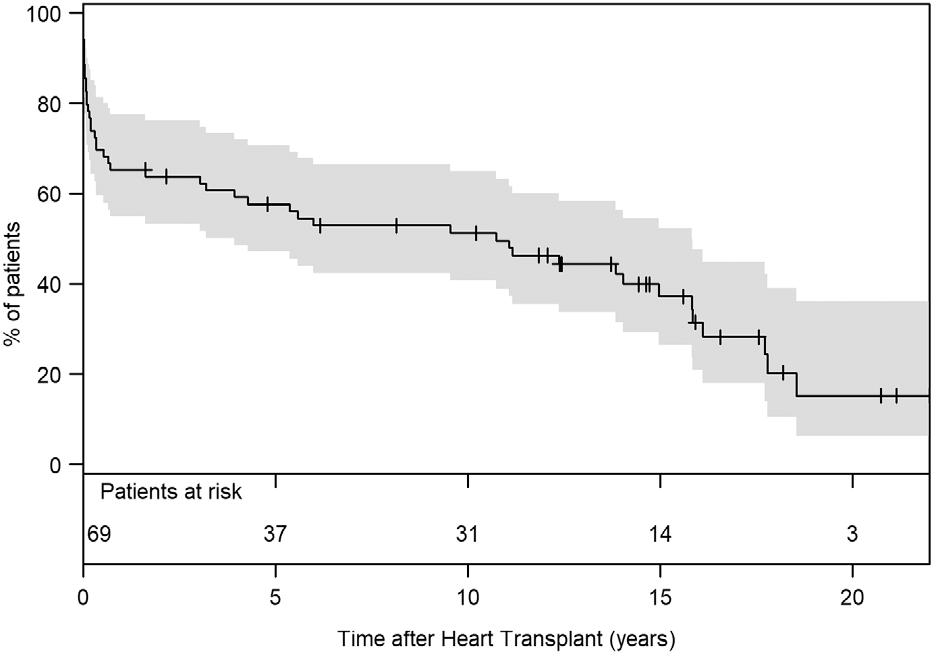
Survival after heart transplant (censored at death). 95% CI, confidence interval.

Survival to heart transplantation was achieved in 69 (35.2%) patients, with most transplants occurring under high‐urgency status (84%). The median posttransplant survival time was 5.8 years [IQR: 0.2–14.2], with the longest surviving patient at 21.4 years at the time of analysis. The 1‐, 5‐, and 10‐year posttransplant survival rates were 65% (95% CI: 55–77), 58% (95% CI: 47–71), and 51% (95% CI: 41–65), respectively (Figure 3).

Peri‐ and postoperative adverse events are summarized in Table 4. Patients who died while on TAH support experienced significantly higher rates of delayed chest closure due to diffuse bleeding or hemodynamic instability (*p* = 0.048), and higher reoperation rates for early postoperative bleeding or atrial tamponade (*p* = 0.022). In two patients, chest closure was impossible postoperatively, leading to immediate placement on the high‐urgency transplant list. Both had normal BMI and received the 70 cc TAH implant. One died on postoperative day 13, while the other was successfully transplanted on day 39.

**TABLE 4:** Perioperative data and adverse events while TAH support.

Variables	Overall group (%) *n* = 196	Survivors under TAH support *n* = 69 (35.2%)	Died under TAH support *n* = 127 (64.8%)	*p* value
Support time (days)	96 (22.5–227)	265 (152–472)	37 (12–123)	***<0.001***
Bypass time, min	160 (131–184)	150 (121–183)	160 (136–184)	0.283
Aortic clamp time, min	121 (107–144)	119 (106–153)	124 (109–140)	0.581
Device type, *n* (%)				
50 cc	6 (3.1)	2 (2.9)	4 (3.1)	0.644
70 cc	190 (96.9)	67 (97.1)	123 (96.9)	
Thorax apertus, *n* (%)	104 (53.1)	30 (43.5)	74 (58.3)	***0.048***
Rethoracotomy, *n* (%)	87 (44.4)	23 (33.3)	64 (50.4)	***0.022***
Postoperative vv‐ECMO, *n* (%)	24 (12.2)	2 (2.9)	22 (17.3)	***0.003***
Postoperative renal replacement therapy, *n* (%)	156 (86.7)	43 (72.9)	113 (93.4)	***<0.001***
Postoperative liver dialysis, *n* (%)	87 (47.8)	19 (30.6)	68 (56.7)	***<0.001***
Postoperative tracheotomy, *n* (%)	105 (55.6)	21 (32.3)	84 (67.7)	***<0.001***
Neurologic event, *n* (%)	77 (39.3)	23 (33.3)	54 (42.5)	0.208
Mesenteric ischemia, *n* (%)	59 (30.3)	9 (13)	50 (39.7)	***<0.001***
Gastrointestinal bleeding, *n* (%)	48 (24.6)	11 (15.9)	37 (29.4)	***0.037***
Retroperitoneal hematoma, *n* (%)	9 (4.6)	1 (1.4)	8 (6.3)	0.164
Abdominal surgery, *n* (%)	41 (21)	8 (11.6)	33 (26.2)	***0.017***
Driveline infection, *n* (%)	12 (6.1)	8 (11.6)	4 (3.1)	***0.027***
Device infection/Mediastinitis, *n* (%)	10 (5.1)	6 (8.7)	4 (3.1)	0.170
Sternal wound infection, *n* (%)	14 (7.1)	8 (11.6)	6 (4.7)	0.087
Pleural empyema, *n* (%)	1 (0.5)	0	1 (0.8)	1.0
Rupture of membrane, *n* (%)	6 (3.1)	2 (2.9)	4 (3.1)	0.644

*Note*: Values are presented as median and interquartile ranges 25th–75th or n (%).

Abbreviations: TAH, total artificial heart; vv‐ECMO, venovenous extracorporeal membrane oxygenation.

Patients who died on TAH support required more frequent postoperative venovenous extracorporeal membrane oxygenation (*p* < 0.003) and tracheotomy (*p* < 0.001) due to respiratory failure and prolonged mechanical ventilation, as well as renal replacement therapy (*p* < 0.001) and liver dialysis (*p* < 0.001). They also experienced more gastrointestinal bleeding (*p* = 0.037) and mesenteric ischemia following open abdominal surgery (*p* < 0.001). A total of 77 patients (39.3%) experienced neurological events, with no significant difference in incidence between groups (*p* = 0.208). All the patients had neurologic physical limitations of varying degrees, except for three patients who had transient ischemic attacks. In 18 patients (23.4%), a neurologic event was the cause of death while on TAH, mostly due to intracranial hemorrhage (14 patients). Another 23 patients (29.8%) with neurologic events were transplanted.

Mediastinitis occurred in 10 patients, with one requiring repeated drainage and irrigation surgeries and another developing pleural empyema requiring surgical sanitation. Driveline infections were more common among survivors (*p* = 0.009) and often led to high‐urgency heart transplantation.

Technical or procedural complications occurred in 12 (6%) patients. In one case, obstruction of the mechanical tricuspid valve of the TAH by a migrated central venous catheter led to death. Six patients experienced membrane rupture of an artificial ventricle; two of these were successfully transplanted, while the third had a successful right ventricle replacement on the 880th day of support. Three patients with driveline tear were also successfully transplanted. Additionally, one patient developed TAH‐induced hemolysis, and another experienced hemodynamically significant narrowing of the pulmonary veins due to the TAH. Both were placed on the high‐urgency transplant list and subsequently transplanted.

#### 3.2.2 Preoperative risk factors for mortality under TAH support

In the univariate Cox regression analysis of perioperative variables, several factors were significantly associated with mortality while on TAH support. These included older patient age, blood groups O and A, elevated levels of alkaline phosphatase and total bilirubin, and the presence of preoperative diagnoses such as acute myocardial infarction, postcardiotomy heart failure, or primary diagnoses from the subgroup labeled “Other indication.” However, subsequent multivariate Cox regression analysis revealed that older patient age, higher total bilirubin levels, a postcardiotomy heart failure, and specific underlying diagnoses from the subgroup “Other indication” were the only independent predictors of mortality during TAH support (Table 5). Additionally, the presence of a preoperative intra‐aortic balloon pump prior to TAH implantation was identified as a factor associated with favorable outcomes.

**TABLE 5:** Results of multivariate Cox regression analysis.

Variables	HR (95% CI)	*p‐*value
Age	1.027 (1.005–1.049)	***0.014***
Preoperative IABP	0.582 (0.376–0.898)	***0.015***
Total bilirubin	1.072 (1.025–1.121)	***0.003***
Postcardiotomy heart failure	2.421 (1.344–4.363)	***0.003***
Diagnosis group “Other indication”	1.963 (1.099–3.505)	***0.023***
Acute myocardial infarction	1.043 (0.611–1.779)	0.877
Alkaline phosphatase	1.002 (0.999–1.005)	0.152
Blood group 0	1.412 (0.639–3.118)	0.393
Blood group A	2.082 (0.957–4.532)	0.065

*Note*: Diagnosis group “Other indication” includes: acute myocarditis, infective endocarditis, cardiac graft rejection, congenital cardiac disease, primary pulmonary hypertension, and cardiac tumor.

Abbreviations: CI, confidence interval; HR, hazard ratio; IABP, intra‐aortic balloon pump.

#### 3.2.3 Survival under TAH support dependent on the primary diagnosis

**FIGURE 4:**
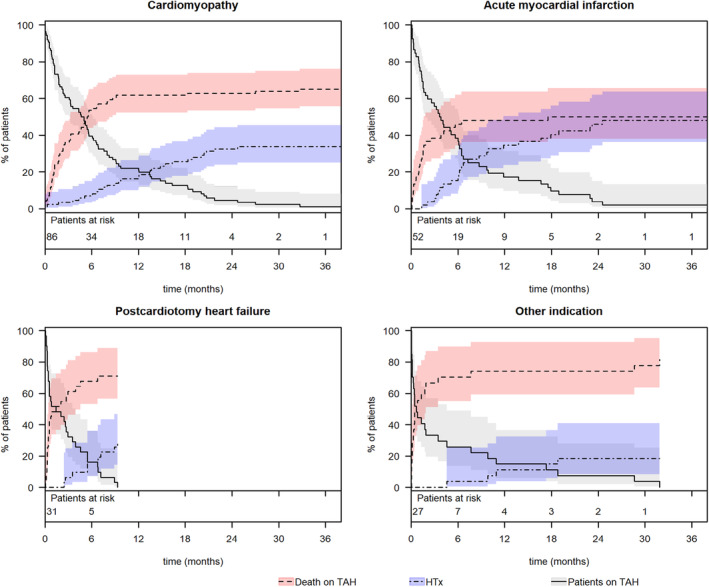
Cumulative Incidence of death and transplantation dependent on the primary diagnoses. 95% CI, confidence interval; TAH, total artificial heart. Diagnosis group “Other indication” includes: Acute myocarditis, infective endocarditis, cardiac graft rejection, congenital cardiac disease, primary pulmonary hypertension, and cardiac tumor. [Color figure can be viewed at wileyonlinelibrary.com]

We noted statistical differences in the cumulative incidence of mortality rates under TAH support (*p* < 0.001) and heart transplant rates (*p* = 0.03) among groups based on their primary diagnosis (Figure 4). A significant disparity was observed in mortality rates under TAH support between patients with postcardiotomy heart failure and those with acute myocardial infarction (71% vs. 50%; *p* = 0.03). Additionally, mortality rates under mechanical support were significantly different in patients from the “Other indication” subgroup compared to those with cardiomyopathies (82% vs. 66%; *p* = 0.001) and acute myocardial infarction (82% vs. 50%; *p* = 0.002). Notably, all patients with infective endocarditis and congenital cardiac disease in the “Other indication” subgroup died while on TAH support. The last of these patients was ineligible for a heart transplant due to immunologic contraindications and nicotine abuse, and died on the 945th day of support. Furthermore, one patient with primary pulmonary hypertension and another with a cardiac sarcoma also died after 2 and 849 days of mechanical support, respectively.

The highest transplant rates were observed in patients primarily suffering from acute myocardial infarction, followed by those with cardiomyopathies (50% vs. 34%, *p* = 0.04). Significantly lower transplant rates were noted for patients with postcardiotomy heart failure (29%) and in the “Other indication” group (18%).

## 4 DISCUSSION

Patients with biventricular failure continue to exhibit high mortality rates. According to the recent STS INTERMACS 2022 Annual Report, the 1‐year survival rate on TAH support was 52.2%.^9^ In our patient collective, the 1‐, 6‐, and 12‐month survival rates under TAH support were 72%, 41%, and 34%, respectively. These rates align with those reported by the European Registry for Patients with Mechanical Circulatory Support (EUROMACS),^14^ but are notably lower than several other previous reports.^15^, ^16^, ^17^, ^18^ Each center has its own implantation criteria, making patient cohorts not always comparable. We broadened our inclusion criteria to implant the TAH even in critically ill patients who were potential transplant candidates. Most of our patients were categorized as INTERMACS Profile 1 or 2, with a high preoperative incidence of cardiac surgery, cardiopulmonary resuscitation, mechanical ventilation, and mechanical circulatory support. Unlike Copeland et al.,^19^ who reported high survival rates in a nonrandomized prospective study, our patient cohort included those with higher bilirubin levels (up to 24.2 mg/dL), many undergoing renal dialysis and various preoperative circulatory support devices. An analysis of adverse events under TAH support in our collective revealed a high incidence of repeat surgeries for substantial early postoperative bleeding or atrial tamponade. Additionally, we often had to delay sternal closure with temporary mediastinal compression packing due to fitting issues or until perioperative bleeding stabilized. We suspect that underlying liver dysfunction in severe biventricular dysfunction, and a high pre‐implant rate of mechanical circulatory support, might contribute to significant coagulopathies. Although the incidence of neurologic events during TAH support was high, we could not always determine the exact timing of stroke onset in some patients. Despite the transcutaneous pneumatic drive lines, repeated surgeries, and extended mean support time, we observed a low incidence of TAH‐associated local infectious complications and device malfunctions. The previously reported fatal iatrogenic device malfunction, caused by the obstruction of the mechanical tricuspid valve of the TAH due to the central venous catheter, has not recurred anymore.^13^, ^20^ The predominant cause of death in our experience was irreversible multiple organ failure that preexisted before TAH implantation.

Our study identified older age, higher total bilirubin level, and underlying diagnoses such as postcardiotomy heart failure as significant and independent risk factors for mortality while on TAH support. These findings echo other reports that have identified older age and higher preimplantation bilirubin levels as mortality risk factors.^17^, ^18^, ^21^, ^22^, ^23^ Unlike Kirsch et al.,^21^ who found preoperative mechanical ventilation as a risk factor for death, we did not observe this association in our patients, possibly because 78.1% of our patients were mechanically ventilated preoperatively, compared to 33% in Kirsch et al.'s study. Intriguingly, postcardiotomy heart failure as the primary diagnosis was also identified as a risk factor for mortality under TAH support. This subgroup experienced exceptionally high mortality and low transplant rates, suggesting they were more critically ill and decompensated preoperatively.

The bridge‐to‐transplant success rate of 35.2% in our study is notably lower than the rates (61–87%) reported in other studies.^15^, ^18^, ^19^, ^21^, ^23^, ^24^, ^25^, ^26^, ^27^ For instance, a retrospective study by Coyan et al.^27^ analyzing heart transplantation listings in the United Network for Organ Sharing (UNOS) database from 2004 to 2020 found that 87% of patients with TAH implants were successfully transplanted. Similarly, Itagaki et al.^23^ reported a 69% transplant success rate in a study of the UNOS system from 2005 to 2018. However, these findings might be overestimated due to the exclusion of patients with a TAH system who were not listed for heart transplantation. Carrier et al.^18^ in their multicenter analysis of 217 patients undergoing TAH implantation in six high‐volume North American centers, reported a 64% heart transplantation rate with a posttransplant survival rate of 84% at 1‐year follow‐up. A meta‐analysis including three studies with 149 TAH patients indicated survival rates after heart transplantation at 1, 5, and 10 years of 83%, 69%, and 55%, respectively.^28^ Our previous reports show comparable 1‐, 5‐, and 10‐year posttransplant survival rates at 64%, 51%, and 50%.

Comparing outcomes and transplant rates between UNOS countries and Europe poses challenges due to differences in allocation systems, donor organ availability and quality. Notably, waiting times for transplants in patients on mechanical support differ significantly between Europe and the United States. Coyan et al. reported an average waiting period of 4.0 months on TAH before the 2018 changes in UNOS allocation, which decreased to 2.8 months afterward.^27^ In contrast, our transplanted patients experienced a median TAH support duration of 265 days, considerably longer than the UNOS‐reported duration. Under UNOS policies, TAH patients were prioritized on the waiting list over others.^29^ However, in Germany, Eurotransplant rules do not prioritize patients with devices, except in cases with device‐related life‐threatening complications. Notably, 84% of our transplanted patients had high‐urgency status. De By et al. also highlighted the relatively low heart transplant rate in EUROMACS‐participating countries, averaging 4.3 transplants per million inhabitants, compared to the US rate of 10.9 per million.^30^ The STS INTERMACS Report 2020 indicates higher heart transplant rates in the United States at 1‐, 3‐, and 5‐year intervals (14.3%, 28.3%, and 32.2%, respectively) as opposed to EUROMACS rates (7.5%, 20.2%, and 25.3%).^8^

## 5 LIMITATIONS

Our study, being non‐randomized and observational in nature, relies on retrospective data collection. The self‐reported results, without external validation from independent data sources, are subject to potential selection bias, which inherently limits the generalizability of our findings.

## 6 CONCLUSION

Despite the widespread development and use of various mechanical circulatory support devices, the SynCardia® temporary TAH remains the device of choice for biventricular support in patients for whom other devices are unsuitable. Considering the extremely poor preoperative clinical status of our patients, many of whom were expected not to survive, we view it as a positive outcome that a third of these patients were successfully bridged to transplantation.

## AUTHOR CONTRIBUTIONS

Artyom Razumov: Conceptualization, data curation, formal analysis, investigation, methodology, project administration, resources; writing—original draft, and writing—review and editing. Melchior Burri: Formal analysis, methodology, software, and writing—review and editing. Armin Zittermann: Formal analysis and writing—review and editing. Darko Radakovic: Formal analysis and writing—review and editing. Volker Lauenroth, Sebastian V. Rojas, Henrik Fox, and Rene Schramm: Writing—review and editing. Jan Gummert: Project administration, supervision, and writing—review and editing. Marcus‐André Deutsch: Conceptualization, data curation, methodology, supervision, writing—original draft, and writing—review and editing. Michiel Morshuis: Conceptualization, project administration, supervision, writing—review and editing. All authors contributed to the writing and editing of the manuscript.

## FUNDING INFORMATION

None.

## CONFLICT OF INTEREST STATEMENT

The authors have no conflicts of interest to declare with respect to this research.
